# A comparative study of bronchodilator response: utilizing pre-bronchodilator versus predicted normal values

**DOI:** 10.1186/s12890-024-02859-4

**Published:** 2024-01-25

**Authors:** Afe Alexis, Naresh M. Punjabi, Kyle Grealis, Adam Wanner

**Affiliations:** Division of Pulmonary, Critical Care, and Sleep Medicine, Department of Medicine, University of Miami, Miller School of Medicine, 1951 NW 7th Avenue, 33146 Miami, FL USA

**Keywords:** Bronchodilator response, Spirometry, Pulmonary function testing

## Abstract

**Background:**

A positive bronchodilator response has been defined as a 12% increase in the forced expiratory volume in one second (FEV_1_) or forced vital capacity (FVC) from their respective pre-bronchodilator values, combined with at least a 0.2 L absolute change. Recent recommendations suggested the use of the percent change in FEV_1_ and FVC relative to their predicted normal values without having applied them in patients with airflow obstruction. The aim of the current study was to compare the two approaches over a wide range of pre-bronchodilator FEV_1_ and FVC values.

**Methods:**

A retrospective review of consecutive patients undergoing spirometry and bronchodilator testing was completed. The change in FEV_1_ and FVC with a bronchodilator was expressed relative to the pre-bronchodilator and predicted normal FEV_1_ and FVC.

**Results:**

In 1,040 patients with a non-paradoxical change in FEV_1_, 19.0% had a ≥ 12% change in FEV_1_ using their pre-bronchodilator value compared to 5.7% using their predicted normal value. For FVC, the respective values were 12.7% vs. 5.8%. The difference was retained in patients with a ≥ 0.2 L change in FEV_1_ or FVC. In unobstructed patients, the upper threshold (two standard deviations above the mean) of the bronchodilator response was 14% for FEV_1_ and 10% for FVC using predicted normal values.

**Conclusions:**

Expressing the percent change in FEV_1_ and FVC relative to predicted normal values reduces the over-estimation of the bronchodilator response, especially in patients with a very low pre-bronchodilator FEV1, including in those with a ≥ 0.2 L change in FEV_1_. Irrespective of pre-bronchodilator values, a ≥ 14% change in FEV_1_ and ≥ 10% change in FVC relative to the predicted normal values could be considered a positive bronchodilator response.

## Background

In pulmonary function testing, the evaluation of a bronchodilator response (BDR) during spirometry involves the administration of inhaled short-acting airway smooth muscle relaxing agents, such as β2-adrenergic agonists. The previous criteria for identifying a positive BDR, as outlined by the American Thoracic Society (ATS) and European Respiratory Society (ERS) guidelines, necessitate both a 0.2 L and 12% increase in forced expiratory volume in one second (FEV1) or forced vital capacity (FVC) [1–3]. If these dual criteria are not met, the BDR is deemed negative. However, it is important to note that this definition of a BDR lacks sensitivity, particularly for individuals with either low or high pre-bronchodilator FEV1 or FVC values.

**Fig. 1:**
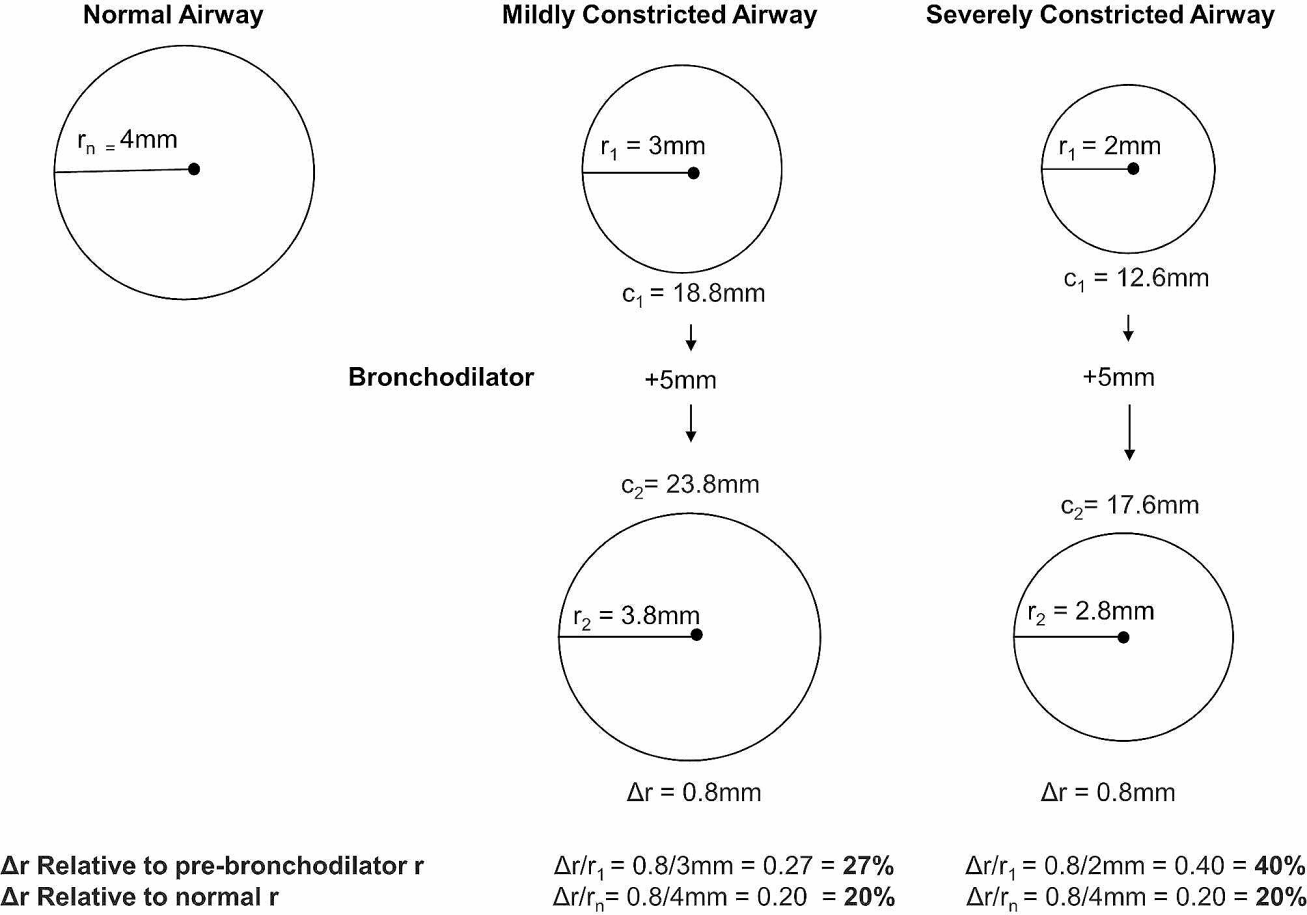
Effects of referencing a bronchodilator-induced increase in airway radius (r) relative to the pre-bronchodilator radius (r_1_ and r_2_) or the radius of a normal airway (r_n_) in two constricted airways

Airflow resistance is inversely related to the airway radius (r) which, in turn, determines airway circumference (c = 2πr). Because bronchodilators decrease airflow resistance by lengthening circumferential airway smooth muscle, any increase in airway smooth muscle circumference can be converted to a related change in the radius (r). For example, a bronchodilator-induced 5 mm increase (Fig. 1) in airway circumference (c) elicits distinct effects on radial change (Δr) when referenced to the baseline radius of the constricted airway (r_1_ or r_2_) or to the radius of a normal airway (r_n_). Consequently, a 5 mm circumference lengthening results in a 27% and 40% radial increase relative to r_1_ (3 mm) and r_2_ (2 mm), respectively. Conversely, when referenced to the normal airway radius (4 mm), the same circumference lengthening yields a 20% radial increase for both airways. Similarly, in the clinical arena, bronchodilator-induced changes in FEV_1_ and FVC, which are surrogates of airway caliber, are exaggerated when referencing to pre-bronchodilator values versus the predicted normal values.

Recently, an ERS/ATS task force revisited the existing ERS and ATS guidelines and recommended a refinement in the assessment of a BDR [4]. The task force proposed normalizing BDR by expressing the changes in FEV_1_ and FVC relative to their predicted normal values [∆FEV_1(%PN)_ and ∆FVC_(%PN)_] to account for differences in pre-bronchodilator FEV_1_ or FVC [4]. Based on information on the BDR in a large cohort of healthy subjects, the task force defined a positive BDR as a ∆FEV_1(%PN)_ and ∆FVC_(%PN)_ of > 12% and > 10%, respectively [5]. Notably, this approach has yet to be applied in patients with airflow obstruction. Leveraging a cohort of patients undergoing pulmonary function testing at a tertiary academic medical center, the current study sought to discern the extent to which utilizing ∆FEV_1(%PN)_ and ∆FVC_(%PN)_ would mitigate bias induced by pre-bronchodilator FEV_1_ and FVC values in the assessment of a positive BDR. Additionally, this study also aimed to establish thresholds for a positive BDR, focusing on a subset of patients with pre-bronchodilator FEV_1_ and FVC values in the normal range.

## Methods

### Study sample and spirometry testing

The study sample consisted of sequential patients who underwent spirometry with bronchodilator testing at the Pulmonary Function Laboratory of the University of Miami Hospital from February 1, 2008, to November 30, 2021 (*N* = 1,637). A deidentified dataset was extracted from the pulmonary function laboratory after the study received approval from the local Institutional Review Board. Patients exhibiting a paradoxical bronchodilator response (ΔFEV1 ≤ 0 L) were excluded. Spirometry was performed by trained technicians according to the pulmonary laboratory protocols per ATS standards. Before commencing the spirometry testing, patients were given comprehensive instructions and a demonstration to ensure a proper understanding of the technique. The Vyntus BODY pulmonary function system (Vyaire Medical, Mettawa, Illinois, USA) which incorporates spirometry with measurements of functional residual capacity and diffusing capacity of the lung was used. All pulmonary function testing took place in the seated position. The standardized sequence for each forced expiratory maneuver involved tidal breathing, maximal inspiration, maximum expiration, and maximal inspiration. Technicians made up to six attempts to acquire three acceptable sets, and the set with the highest FEV_1_ was selected for analysis. Real-time error detection during maneuvers prompted immediate technician intervention, aligning with ATS recommendations. Spirometry assessments were conducted both before and 10 min after the administration of 2.5 mg/3 ml albuterol solution via a jet nebulizer, with a nebulization duration of 5–7 min. Informed consent was waived by the University of Miami Institutional Review Board given the retrospective review of de-identified data.

### Statistical analysis

To assess and compare the BDR in FEV_1_ and FVC relative to the pre-bronchodilator or predicted normal values, bivariate scatter plots were used. Subgroup analyses on BDR were undertaken in a subset of patients with a Δ0.2 L change in FEV_1_ (*N* = 213). Comparisons of ∆FEV_1(%Pre−B)_ to %ΔFEV_1(%PN)_ and ∆FVC_(%Pre−B)_ to %ΔFVC_(%PN)_ were conducted as a function of the pre-bronchodilator FEV_1_ in all patients and the subset of patients with a ≥ 0.2 L change in FEV_1_. In addition, analyses were also conducted in a subgroup of patients with a pre-bronchodilator FEV_1_ or FVC > 80% of predicted normal (*N* = 462). All analyses were conducted using Stata 17.0 (Stata Corp, College Station, TX).

## Results

### Sample characteristics

Table 1 presents the demographic and spirometry data on the full sample and associated patient subsets. Of the initial 1,637 patients who underwent both pre- and post-bronchodilator spirometry, 1,040 exhibited a ΔFEV_1_ > 0 L, and 928 had a ΔFVC > 0 L following the bronchodilator challenge. Within the subset of patients with a ΔFEV_1_ > 0 L, 213 demonstrated a ≥ 0.2 L change in FEV_1_. Moreover, among the 1,637 patients, 462 displayed pre-bronchodilator FEV_1_ and FVC values exceeding 80% of predicted normal values.

**Table 1:** Characteristic of Study Samples

	All Patients		ΔFEV_1_ > 0.0 L		ΔFEV_1_ ≥ 0.2 L		ΔFVC > 0.0 L		Pre-FEV_1_ > 80% Pre-FVC > 80%	
N	1637		1040		213		928		462	
Age*	59.7	(16.2)	59.2	(16.2)	55.9	(16.9)	59.3	(16.4)	55.1	(17.4)
Male sex	787	(48.1%)	503	(48.4%)	141	(66.2%)	452	(48.7%)	182	(39.4%)
Race										
Hispanic	814	(49.7%)	510	(49.0%)	99	(46.5%)	264	(28.5%)	218	(47.2%)
White	475	(29.0%)	308	(29.6%)	76	(35.7%)	464	(50.0%)	143	(31.0%)
Black	312	(19.1%)	197	(18.9%)	35	(16.4%)	180	(19.4%)	94	(20.4%)
Other	36	(2.2%)	25	(2.4%)	3	(1.4%)	20	(2.2%)	7	(1.5%)
FEV_1_, L*	1.9	(0.8)	1.9	(0.8)	2.3	(0.9)	1.9	(0.8)	2.7	(0.8)
FVC, L*	2.8	(1.1)	2.8	(1.1)	3.4	(1.2)	2.7	(1.0)	3.5	(1.0)
FEV1/FVC%*	69.1	(14.5)	68.8	(14.6)	66.8	(13.2)	68.6	(15.0)	76.8	(7.6)

^*^ Values represent mean (SD)

### BDR based on the FEV_1_

**Fig. 2:**
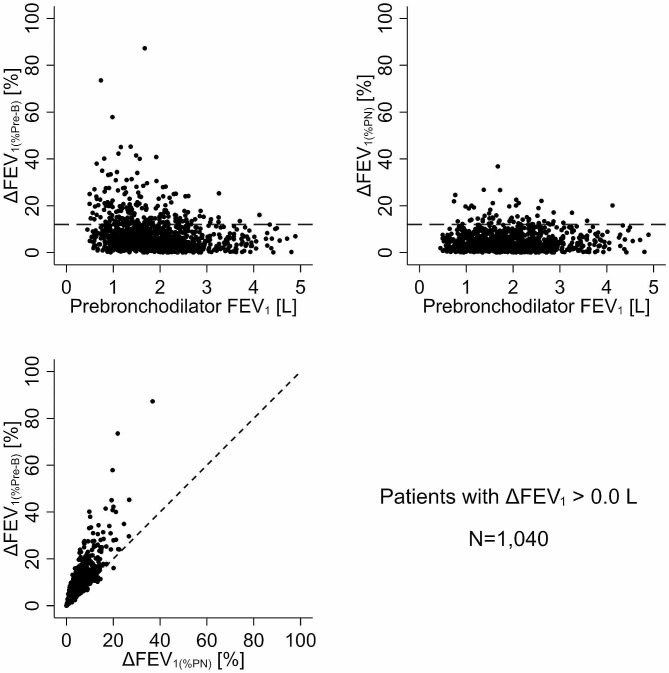
Plot of bronchodilator-induced change in FEV_1_ relative to pre-bronchodilator FEV_1_ (∆FEV_1(%Pre−B)_; top left panel) and relative to predicted normal FEV_1_ (∆FEV_1(%PN)_; top right panel) as a function of pre-bronchodilator FEV_1_ in patients with a positive change in FEV_1_ (*N* = 1,040). The horizontal dashed line indicates a 12% change. The bottom left panel is the scatter plot of ∆FEV_1(%Pre−B)_ vs. ∆FEV_1(%PN)_ with the diagonal dashed line as the line of identity.

Figure 2 illustrates the bivariate scatter plots for pre-bronchodilator FEV_1_, ∆FEV_1(%Pre−B)_ and ∆FEV_1(%PN)_. Regardless of the method used to reference the change in FEV_1_ following bronchodilator administration, a considerable proportion of patients with a low pre-bronchodilator FEV_1_ had a > 12% change indicative of a positive BDR. Employing the traditional definition with pre-bronchodilator FEV_1_ as the reference, 19.0% of patients (95% CI: 16.6-21.6%) met the BDR criterion. In contrast, when using the predicted normal FEV_1_ as the reference, only 5.7% of patients (95% CI: 4.3-7.3%) had a positive BDR. Consequently, a quantitative disparity emerged with ∆FEV_1(%Pre−B)_ classifying approximately 14% more patients as having a BDR than with ∆FEV_1(%PN)_ (*p* < 0.001). The scatterplot of ∆FEV_1(%Pre−B)_ versus ∆FEV_1(%PN)_ revealed that 93.1% of patients fell above the line of identity, confirming a systematic difference between the two approaches for referencing the ∆FEV_1_ with a bronchodilator (Fig. 2; bottom left).

**Fig. 3:**
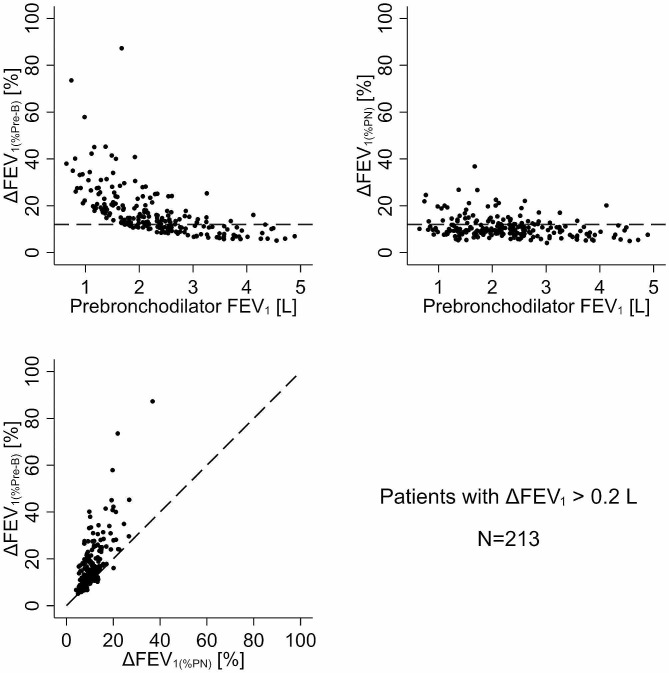
Plot of bronchodilator-induced change in FEV_1_ relative to pre-bronchodilator FEV_1_ (∆FEV_1(%Pre−B)_; top left panel) and relative to predicted normal FEV_1_ (∆FEV_1(%PN)_; top right panel) as a function of pre-bronchodilator FEV_1_ in patients with a ΔFEV_1_ ≥ 0.2 L (*N* = 213). The horizontal dashed line indicates a 12% change. The bottom left panel is the scatter plot of ∆FEV_1(%Pre−B)_ vs. ∆FEV_1(%PN)_ with the diagonal dashed line as the line of identity

In the subset of patients (*N* = 213) with a ≥ 0.2 L change in FEV_1_, the disparity in the proportion of patients with a positive BDR persisted when comparing ∆FEV_1(%Pre−B)_ to ∆FEV_1(%PN)_. Using the ∆FEV_1(%Pre−B)_ to define a positive BDR, 64.3% of patients (95% CI: 57.5-70.7%) exceeded the 12% threshold (Fig. 3; top left), in contrast to 25.8% (95% CI: 20.2-32.2%) when using ∆FEV_1(%PN)_ to define a positive BDR (Fig. 3; top right). Consequently, compared to ∆FEV_1(%PN)_, the number of patients with a > 12% BDR remained 40.4% higher when using ∆FEV_1(%Pre−B)_ while also requiring a ≥ 0.2 L change in ∆FEV_1_. The difference in the proportion of BDR-positive patients based on the two references was visually evident in the scatterplot of ∆FEV_1(%Pre−B)_ vs. ∆FEV_1(%PN)_, where 90.6% of patients were above the line of identity (Fig. 3; bottom left).

### BDR based on the FVC

**Fig. 4:**
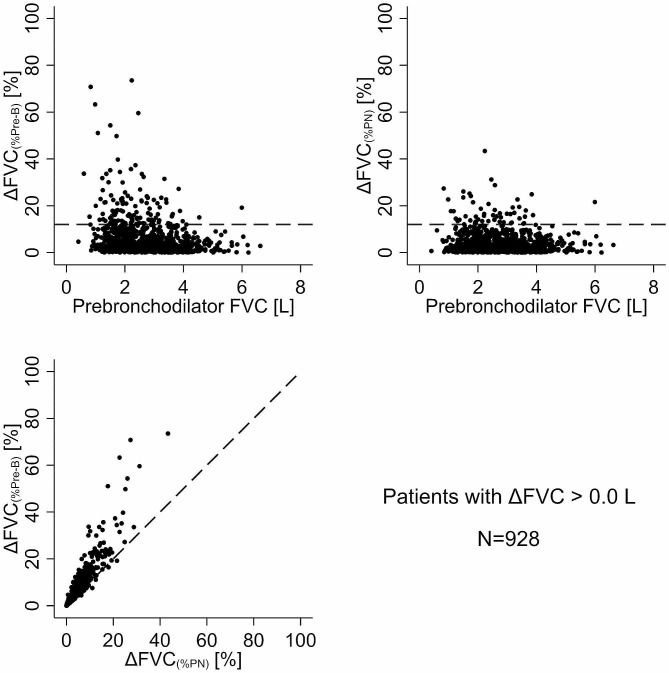
Plot of bronchodilator-induced change in FVC relative to pre-bronchodilator FVC (∆FVC_(%Pre−B)_; top left panel) and relative to predicted normal FVC (∆FVC_(%PN)_; top right panel) as a function of pre-bronchodilator FVC in patients with a ∆FVC ≥ 0.0 L (*N* = 928). The horizontal dashed line indicates a 12% change. The bottom left panel is the scatter plot of ∆FVC_1(%Pre−B)_ vs. ∆FVC_(%PN)_ with the diagonal dashed line as the line of identity

**Fig. 5:**
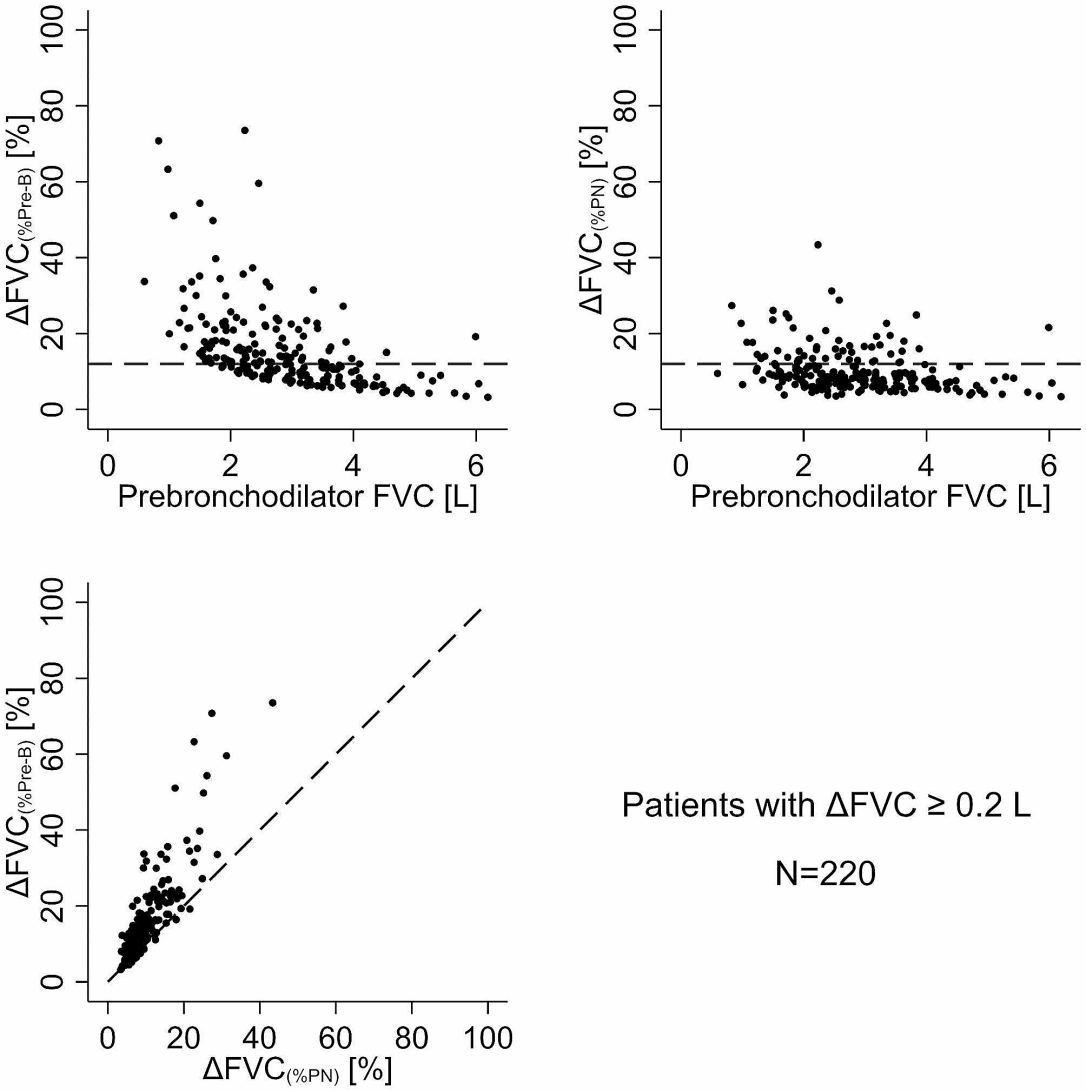
Plot of bronchodilator-induced change in FVC relative to pre-bronchodilator FVC (∆FVC_(%Pre−B)_; top left panel) and relative to predicted normal FVC (∆FVC_(%PN)_; top right panel) as a function of pre-bronchodilator FVC in patients with a ∆FVC ≥ 0.2 L (*N* = 220). The horizontal dashed line indicates a 12% change. The bottom left panel is the scatter plot of ∆FVC_1(%Pre−B)_ vs. ∆FVC_(%PN)_ with the diagonal dashed line as the line of identity

A discernible systematic difference also emerged between the two methods of assessing BDR in the proportion of patients when referencing the change in FVC to the pre-bronchodilator value [∆FVC_(%Pre−B)_] versus the predicted normal FVC value [%∆FVC_(%PN)_]. Within the cohort of 928 patients exhibiting a non-paradoxical ∆FVC > 0 L, 12.7% of patients (95% CI: 10.6–15.0%) surpassed the 12% BDR threshold using ∆FVC_(%Pre−B)_ (Fig. 4; top left) compared to 5.8% (95%CI: 4.4-7.5%) for %∆FVC_(%PN)_ (Fig. 4; top right). The scatterplot comparing ∆FVC_(%Pre−B)_ to ∆FVC_(%PN)_ revealed that 70.8% of patients were above the line of identity (Fig. 4; bottom left). Even among the patients with a ≥ 0.2 L change in FVC, 51% demonstrated a > 12% change in ∆FVC_(%Pre−B)_ compared to 25% for %∆FVC_(%PN)_**(**Fig. 5). The ∆FVC_(%Pre−B)_ vs. ∆FVC_(%PN)_ analyses underscored this difference, with 90% of patients falling above the line of identity (Fig. 5; bottom left).

### BDR in patients with a pre-bronchodilator FEV_1_ and FVC > 80% of predicted normal

Within the cohort of 1,040 patients demonstrating a ΔFEV_1_ > 0 L, a subset of 462 patients exhibited a pre-bronchodilator FEV_1_ and FVC > 80% of predicted normal (Table 1). In this subgroup, the raw mean predicted normal FEV_1_ was at 94.9%, and mean predicted normal FVC was 97.6%. The average ∆FEV_1(%PN)_ was 5.0% (SD: 4.3), while the corresponding mean ∆FVC_(%PN)_ was 3.2% (SD: 3.4). Employing two standard deviations above the mean as a threshold, a ∆FEV_(%PN)_ of ≥ 14% and ∆FVC_(%PN)_ of ≥ 10% could be considered the thresholds for a positive BDR irrespective of the pre-bronchodilator FEV_1_ or FVC.

## Discussion

The results of this study underscore that normalizing the bronchodilator-induced change in FEV_1_ and FVC relative to the predicted normal FEV_1_ and FVC values reduces the proportion of patients exhibiting a positive BDR, compared to referencing their prebronchodilator values. This reduction in BDR is particularly noteworthy in patients with a low pre-bronchodilator FEV_1_, even among those with a ≥ 0.2 L absolute change in FEV_1_. Regardless of pre-bronchodilator values, a ≥ 14% change in FEV_1(%PN)_ and ≥ 10% in FVC_(%PN)_ could be considered a positive BDR. Expressing the changes in FEV_1_ and FVC relative to predicted normal values addresses the biases introduced by using the pre-bronchodilator FEV_1_ and FVC in the assessment of the BDR (1–3). Additionally, this approach compensates for inter-individual differences in predicted normal FEV_1_ and FVC values, addressing a limitation associated with evaluating BDR by a fixed absolute change in FEV_1_ or FVC [6].

The misclassification in BDR among patients with a low or high pre-bronchodilator FEV_1_ or FVC value has been previously documented in the COPDGene study [6]. In that study, the percent change in FEV_1_ using the pre-bronchodilator values [∆FEV_1(%Pre−B)_] aligned with the absolute change in FEV_1_ (∆FEV_1_) only at a pre-bronchodilator FEV_1_ of approximately 1 L. At that degree of airflow obstruction, ∆FEV_1(%Pre−B)_ and ∆FEV_1_ were 16% and 0.16 L, respectively. As anticipated, these two parameters (i.e., ∆FEV_1(%Pre−B)_ and ∆FEV_1_) diverged significantly at lower and higher pre-bronchodilator FEV_1_ values. The authors of that study suggested that the magnitude of the BDR is best assessed by ∆FEV_1_ and proposed defining a positive BDR as a ∆FEV_1_ > 0.16 L, irrespective of the corresponding ∆FEV_1(%Pre−B)_. However, this approach neglects inter-individual differences in the predicted normal FEV_1_. For example, a ∆FEV_1_ of 0.16 L in a patient with a predicted normal FEV_1_ of 3 L cannot be equated with the same ∆FEV_1_ change in a person with a predicted normal FEV_1_ of 4 L. Furthermore, the data were obtained among patients with COPD, excluding other forms of obstructive lung disease including asthma, limiting the generalizability of the results.

The bias introduced by pre-bronchodilator FEV_1_ and FVC in the grading of the BDR may necessitate a re-definition of what constitutes a positive BDR. As demonstrated in the current study, the conventional ATS/ERS guideline (a 0.2 L and 12% increase in FEV_1_ or FVC) is susceptible to this bias, disproportionately identifying a larger number of severely obstructed patients as having a positive BDR. Hansen et al. [6] suggested that, in grading BDR, a > 0.16 L change in FEV_1_ can be considered positive, irrespective of pre-bronchodilator FEV_1_, based on data from patients with COPD. However, it remains unclear whether this observation can be extrapolated to other patients with airflow obstruction, including asthma. Despite the clinical diagnosis accompanying the request for pulmonary function testing in our study, this information was not utilized due to its poor accuracy. Nevertheless, it was assumed that our dataset, originating from a tertiary care pulmonary function laboratory, included patients with various forms of obstructive lung diseases, including asthma. Therefore, the normalized approach used in BDR assessment is deemed applicable to all patients with airflow obstruction. Another potential limitation of the suggested 0.16 L change in FEV_1_ to define a positive BDR is the oversight of predicted normal FEV_1_ and FVC, as an absolute ∆FEV_1_ and ∆FVC in liters may not account for such inter-individual differences.

The criteria for grading BDR, whether based on FEV_1_ or FVC, and establishing a positive BDR warrant careful consideration. It was reasoned that a positive BDR could be defined as a ∆FEV_1(%PN)_ or ∆FVC_(%PN)_ exceeding values observed in patients with a pre-bronchodilator FEV_1_ or FVC within the predicted normal range. In the subset of patients meeting this criterion (i.e., having a pre-bronchodilator FEV_1_ or FVC > 80% of predicted normal), 14.0% and 10.0% exceeded two standard deviations above the mean for %∆FEV_(%PN)_ %∆FVC_(%PN)_, respectively. Assuming that this BDR reflects reversal of the “normal” bronchomotor tone [7], we propose defining a positive BDR as a ≥ 14% increase in ∆FEV_1(%PN)_ or ≥ 10% increase in ∆FVC_(%PN)_, irrespective of absolute changes in liters or pre-bronchodilator FEV_1_ or FVC. Tan et al. [5] reported BDR in 3,922 healthy subjects and found a mean ∆FEV_1(%PN)_ of 10% (upper 95%CI: 10.5) and 9.2% (upper 95%CI: 10.5) for %∆FVC_(%PN)_. Based on these data, recent ERS/ATS recommendations define a positive BDR as a ∆FEV_1(%PN)_ or ∆FVC_(%PN)_ of > 10% [5]. However, this recommendation has not previously been validated in patients with airflow obstruction. The current study, conducted on a large cohort of patients from a tertiary care setting, demonstrates that applying the normalized BDR method reduces bias introduced by pre-bronchodilator FEV_1_ and FVC. Regarding the definition of a positive BDR, the data herein support a ∆FEV_1(%PN)_ ≥ 14% or ∆FVC_(%PN)_ ≥ 10% in agreement with the recent ERS/ATS standards for FVC but not for FEV_1_ (> 10% for the ERS/ATS and ≥ 14% in the current study). The difference could relate to the respective method of data analysis. The ERS/ATS definitions are based on data obtained in healthy non-smokers irrespective of pre-bronchodilator values whereas we obtained our limits in patients with pre-bronchodilator FEV_1_ and FVC values > 80% of predicted normal, possibly including patients with mild airflow obstruction The application of the recent ERS/ATS standards for assessing the BDR has been tested in patients with established medical diagnoses to understand its impact on clinical practice. Li et al. [8] demonstrated that the new ERS/ATS approach to assessing BDR better differentiated between COPD and asthma than previous ERS/ATS standards. Furthermore, Chaiwong et al. [9] found that in the clinical characterization of obstructive lung disease the previous and recent ERS/ATS standards can be used interchangeably. Finally, Beasley et al. [10] suggested that either definition of a positive bronchodilator response has a minor role in the diagnosis of obstructive lung disease. In contrast, using the recent ERS/ATS standards may have therapeutic implications in children with asthma [11]. Perhaps the definition of a positive bronchodilator response proposed in the current manuscript may be more discriminatory in the classification of patients with obstructive lung disease.

In summary, this study demonstrates the importance of using ∆FEV_1(%PN)_ or ∆FVC_(%PN)_ for assessing the BDR in patients with airflow obstruction, particularly those with lower pre-BD FEV_1_ and FVC values. Unlike the recent ERS/ATS definition of a positive BDR, derived solely from data in clinically normal subjects (5), the current study used a comprehensive pulmonary function dataset encompassing a diverse range of pre-bronchodilator FEV_1_ and FVC values. In contrast to the updated ERS/ATS standards, our findings advocate for raising the threshold for a positive ∆FEV_1(%PN)_ from 10 to 14%, while confirming the 10% for ∆FVC_(%PN)_.

## Data Availability

No datasets were generated or analysed during the current study.
